# Historical and current perspectives on Japanese encephalitis in Sulawesi, Indonesia

**DOI:** 10.14202/vetworld.2025.419-439

**Published:** 2025-02-19

**Authors:** Nur Rahma, Harimurti Nuradji, NLP Indi Dharmayanti, Indrawati Sendow, Rahmat Setya Adji, Muharam Saepulloh, Rusdiyah Rusdiyah, Isra Wahid

**Affiliations:** 1 Research Center for Veterinary Science, National Research and Innovation Agency, Cibinong, 16911, Indonesia; 2 Department of Biomedical Sciences, Postgraduate Program, Hasanuddin University, Makassar, 90245, Indonesia; 3 Research Center for Veterinary Science, National Research and Innovation Agency, Cibinong, 16911, Indonesia; 4 Molecular Biology Research Center, National Research and Innovation Agency, Cibinong, 16911, Indonesia; 5 Center for Zoonotic and Emerging Diseases, Faculty of Medicine, Hasanuddin University, Makassar, 90245, Indonesia

**Keywords:** amplifying hosts, epidemiology, Japanese encephalitis, Sulawesi, vectors, zoonotic reservoirs

## Abstract

Japanese encephalitis (JE), a mosquito-borne viral disease, poses significant public health risks in endemic regions, such as Indonesia. Sulawesi, one of the archipelago’s largest islands, presents a high potential for JE transmission due to its conducive environmental, economic, and cultural factors. Between 1972 and 2017, JE-positive samples were detected sporadically in various hosts, including humans, pigs, bats, cattle, goats, chickens, and mosquitoes (*Culex tritaeniorhynchus*). This review consolidates historical data and provides a contemporary perspective on JE ecology in Sulawesi. The island’s extensive rice fields (95% of districts) and its high density of amplifying hosts – especially pigs, which inhabit 65.5% of districts – highlight critical transmission dynamics. In addition, Sulawesi supports a diverse array of reservoir hosts, such as endemic bats and bird species, which enhance JE’s zoonotic potential. Bats, including *Dobsonia viridis* and *Rousettus celebensis*, are particularly notable for their reservoir roles. Furthermore, at least nine mosquito vector species, led by *C. tritaeniorhynchus*, thrive in Sulawesi’s wetland ecosystems, amplifying transmission risk. Despite the island’s high-risk profile, JE surveillance remains inconsistent, with limited government-led diagnostic programs. Historical and recent data underscore the need for systematic investigations into JE’s epidemiology, emphasizing molecular and serological detection, vector surveillance, and the role of amplifying hosts in transmission cycles. Key challenges include limited awareness, diagnostic infrastructure, and climate change, which exacerbate vector bionomics and disease dynamics. This review advocates for the integration of JE diagnostic tools, public health interventions, and vaccination programs tailored to Sulawesi’s ecological and sociocultural context. These measures are essential to mitigate JE transmission and protect both human and animal health.

## INTRODUCTION

Japanese encephalitis (JE) is caused by the JE virus and is common in many regions of Asia, with an estimated 68,000 clinical cases each year. Although JE symptoms rarely appear, the mortality rate can reach 30%, and permanent neurological and psychiatric effects range from 30% to 50%. Symptoms can be mild (such as fever and headache) or asymptomatic. In children, JE often causes gastrointestinal pain. In severe cases, the disease can lead to disorientation, coma, paralysis, and death. Survivors may experience permanent neurological effects [1, 2]. The neuroinvasive effects are caused by the virus crossing the blood–brain barrier and forming complexes with neuron surface receptors. The combination of neuronal cell death and an uncontrolled neuroinflammatory response leads to high viral pathogenicity [3, 4]. JE primarily affects children, although recent data from Northern China show an increase in cases among adults aged 40 years and older [5].

JE virus belongs to the Flaviviridae family, orthoflavivirus genus. The virion, which has a spherical shape and a diameter of 50 nm, carries a positive-sense ssRNA genome of 9.2–11.0 kb. It comprises three structural proteins – capsid (C), envelope (E), and membrane (M) or preMembrane (prM) in its immature form – alongside seven non-structural proteins: NS1, NS2A, NS2B, NS3, NS4A, NS4B, and NS5. Based on its envelope structure, the JE virus consists of five genotypes: GI, GII, GIII, GIV, and GV. JE is known from the Indomalayan region, where all JE virus genotypes can be found. All genotypes are present in Indonesia except GV, which is found in Malaysia [6–8]. The genotype is important because it influences the genetic diversity of the virus, its behavior, and its response to control measures. GI, GIII, and GIV still exist and are maintained in several Asian countries. Although initially limited to Indonesia, GIII has also been reported in Australia [8–12].

This review consolidates historical and contemporary data on JE in Sulawesi, emphasizing the important roles of extensive rice fields, high pig densities, diverse reservoir hosts, mosquito vectors, and the interplay of sociocultural and economic factors in JE transmission

## JE TRANSMISSION AND RISK FACTORS

JE transmission occurs when the viral pathogen is present in a host capable of amplifying the virus (amplifying host) and, in some cases, facilitating its spread to other locations (migrating host). Subsequently, a carrier agent (vector) is required to transfer the virus to a susceptible host. Vectors are usually insects, primarily mosquitoes that require breeding sites in water-rich areas, such as rice fields, rivers, and swamps [13–15].

The virus multiplies in amplifying hosts, primarily pigs associated with the domestic cycle [16]. Pigs are the most frequently reported hosts of the JE antigen with relatively high infection rates, as previously reported in Jakarta [17], West Kalimantan [18], East Java [19], East Sumba [20], Tangerang [21] and Bali [19, 22]. The host will experience a period of viremia after infection. In pigs, viremia begins on the 1^st^ day, peaks on the 3^rd^ day, and continues to replicate until the 11^th^ day. The virus does not significantly decline despite the presence of a neutralizing antibody response [8, 23–26]. There is clear evidence of high viremia in pigs but no obvious symptoms. The effects of this condition include fever and anorexia, and on a larger scale, it affects pig reproduction, such as the risk of stillbirth and congenital abnormalities [27–29].

Wading birds can also serve as amplifying hosts, especially those from the Ardeidae family, such as egrets and herons. The ability of birds to fly also makes them migratory hosts. Species such as *Ardeola grayii*, *Bubulcus ibis*, *Egretta garzetta*, and *Nycticorax nycticorax* have been studied for their association with JE transmission [30–32]. Other species known to have high titers include ducks, chickens, pigeons, and sparrows. The experiment was carried out by inoculating the JE strain and showed that the virus could be detected in Mallards (*Anas platyrhynchos*), Sparrows (*Passer domesticus*), Red-winged Blackbirds (*Agelaius phoeniceus*), Rock Pigeons (*Columba livia*), European Starlings (*Sturnus vulgaris*), House finch (*Carpodacus mexicanus*), grackle (*Quiscalus quiscula*), ring-billed gull (*Larus delawarensis*), cow egret (*B. ibis*), chicken (*Gallus domesticus*) and great egret (*Ardea alba*). However, the species in this experiment are still considered reservoir-susceptible hosts of the JE virus [33–35].

Other hosts as reservoirs only accommodate the viruses, including bats, cattle, goats, horses, orangutans, and macaques [21, 22, 36–39]. In Malaysia, JE is also detected in birds and cats [40], whereas it is also detected in dogs in Cambodia [41]. In 2018, JE antibody responses were tested in pigs, chickens, ducks, and dogs. The results show that the highest chance of transmission occurs in pigs, but at a certain ratio, the transmission strength in ducks is higher than in pigs, followed by dogs, which have a greater potential than chickens [34].

However, some species are also migratory hosts, such as bats. Because of their abundance, flight capability, and immune resilience, bats can carry various types of viruses, including JE. Bats are second in abundance after rodents, accounting for up to 20% of all mammals. During flight, bats experience physiological temperatures similar to those of fever, which enhance their immune responses. Although the JE virus has been found in brain tissue, no symptoms have been observed. Several bat families from which JE viruses have been isolated include *Pteropodidae*, *Rhinolophidae*, *Hipposideridae*, and *Vespertilionidae*. [42]. In Indonesia, JE has been confirmed to be positive in *Pteropus*, *Cynopterus*, *Eonycteris*, *Hipposideros*, *Kerivoula*, *Macroglossus*, *Pipistrellus*, *Rousettus*, *Scotophilus*, and *Thoopterus* [43, 44]. JE antibodies have been detected in *Rousettus leschenaultia*, *Taphozous melanopogon*, and *Miniopterus fuliginosus* in China [45], while in Yunnan in *Maurina aurata* [46]. In Japan, it was detected in blood and brown fat samples in *Miniopterus schreibersii fuliginosus* and *Rhinolophus cornutus cornutus*, while in Taiwan, it was detected in *Hipposideros armiger terasensis* and *Miniopterus schreibersii fuliginosus* [47, 48]. Genetic and amino acid sequence comparisons have also been performed for *Myotis ricketti*, *Miniopterus schreibersii*, and *Rhinolophus affinis* [49]. The large number of species that have been discovered shows that bats have great potential as reservoirs, especially in close proximity to residential areas.

The virus is transmitted through mosquito bites, and it has been found in 41 types of mosquitoes belonging to the genera *Aedes*, *Anopheles*, *Armigeres*, *Coquillettidia*, *Culex*, *and* *Mansonia*. However, transmission ability depends on the vector competence – mosquito’s intrinsic ability to transfer the virus to another host. Studies have confirmed that there are 17 of the most important species [50, 51], including *Culex tritaeniorhynchus*, *Culex gelidus*, *Culex vishnui*, *Culex fuscocephala*, *Culex bitaeniorhynchus*, *Culex quinquefasciatus*, *Anopheles vagus*, *Anopheles kochi*, *Anopheles annularis*, and *Armigeres subalbatus* [52].

Systemic infection involves the ability of the virus to cross the physiological barrier in the midgut of mosquitoes and spread in the salivary glands. The period from mosquito exposure until it can transmit the virus is referred to as the extrinsic incubation period [53]. In addition, direct transmission without vectors through the saliva of infected pigs can occur. The possibility of sexual transmission to humans has been studied and is possible because the JE virus targets the vaginal epithelium and can survive in the vaginal mucosa for up to 28 days [54].

**Figure 1:**
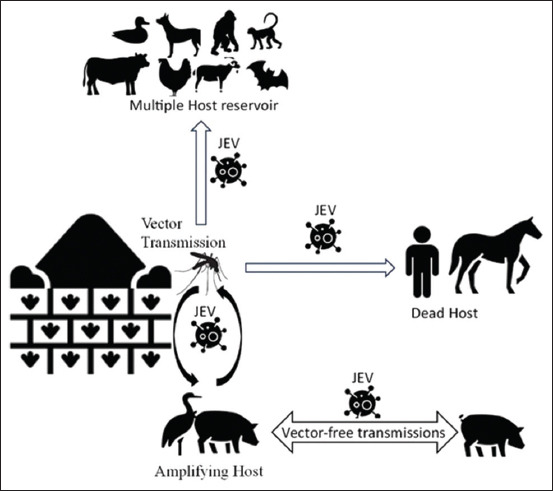
The pattern of Japanese encephalitis virus transmission [Source: Icons from www.flaticon.com].

The virus passes through the subcutaneous layer and undergoes initial replication in the host’s skin cells. Incubation of the virus lasts for 6–16 days and can be resisted directly by the peripheral immune response. If the virus survives, it will continue to spread through hematogenous and efferent lymphatic pathways to the heart, liver, spleen, and central nervous system (CNS) [4, 55]. The JE transmission pattern is shown in Figure 1.

## TOPOGRAPHY-SUPPORTING RISK FACTORS FOR SULAWESI

### Topography

Sulawesi is one of the islands in the Indonesian archipelago that has a very interesting formation history because it is the meeting point between the Eurasian, Pacific, and Australian plates [56]. Sulawesi occupies the eleventh position among the world’s largest islands, boasting remarkable biodiversity. The tropical rainforest characteristics allow for the discovery of various endemic species, although agricultural activities, logging, and mining have adverse effects. Sulawesi is the largest island in the Wallacea region and consists of 6 provinces: Gorontalo (6 districts/cities), South Sulawesi (24 districts/cities), Southeast Sulawesi (7 districts/cities), Central Sulawesi (13 districts/cities), North Sulawesi (15 districts/cities), and West Sulawesi (6 districts), with a total of 81 districts/cities [57, 58]. As a Wallacea region, Sulawesi has a high endemicity of flora and fauna (especially avian and mammals), contributing to species richness and promoting the emergence of diseases, especially zoonotic diseases such as JE.

The primary vector and other known vectors were also found in almost all regions of Sulawesi. The presence of these vectors is closely related to rice fields because rice fields serve as breeding grounds with relatively calm water, providing a food source for larvae and almost no predators. Rice fields in Sulawesi are extensive and are found in 95% of districts/cities (77/81). The largest rice fields are in South Sulawesi, particularly in Bone and Wajo, then in Central Sulawesi (Parigi Moutong), Southeast Sulawesi (Konawe), North Sulawesi (Bolaang Mongondow), West Sulawesi (Polewali Mandar), and the last in Gorontalo province [59–64].

### Risk factor

Risk factors for JE (including pigs, ardeid birds, bats, and mosquito vectors) can be found in nearly all provinces in Sulawesi. Pigs in Sulawesi are reported to be found in 53/81 cities/districts (n = 65.5%). Most commonly found in South Sulawesi, highest in North Toraja and Tana Toraja, followed by West Sulawesi (Mamasa), Central Sulawesi (Konawe), West Sulawesi (Central Mamuju), and North Sulawesi in Minahasa. Meanwhile, Gorontalo has the lowest number of pigs in all provinces [59–64].

**Figure 2:**
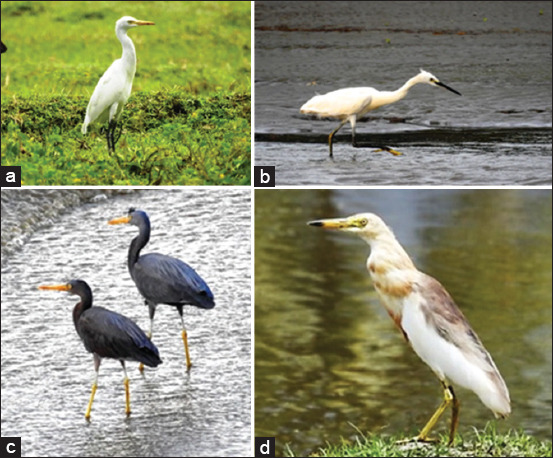
Some Ardeid species occur in Sulawesi, including: (a) *Bubulcus ibis*, (b) *Egretta garzetta*, (c) *Egretta. sacra*, and (d) *Ardeola speciosa*. Photograph taken in Donggala, North Sulawesi [Source: https://komiu.id/].

Ardeid birds are often found along the Sulawesi coast. Commonly encountered species belong to genera such as *Ardea*, *Ardeola*, *Bubulcus*, and *Egretta* (some species in Figure 2). These birds are locally known by various names, such as Kunae, Pakangnggoyo, Ngonae, Timboko, Bonge, Timboko Uba, and Timboko Baula [65]. These birds are present year-round, and their numbers increase during the breeding season. Their ability to fly provides them with a wide range and allows them to move to other locations. Birds are also found in settlements, rivers and lakes, as feeding grounds.

**Figure 3:**
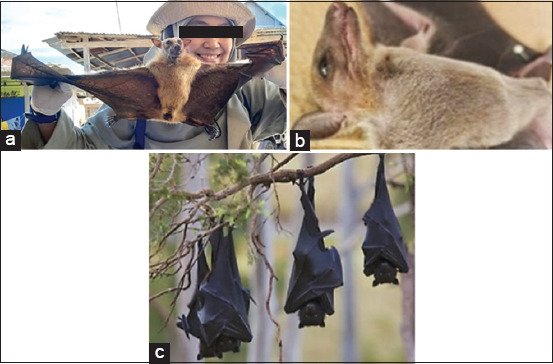
Several bat species occur in Sulawesi, including (a) *Rousettus amplexicaudatus*, (b) *Rousettus. celebensis*, and (c) *Pteropus vampirus*. a. and b. found in Gowa and Maros, South Sulawesi [Source: Center for Zoonotic and Emerging Diseases], while c. from Kolaka, Southeast Sulawesi [source: https://www.inaturalist.org].

Sulawesi harbors significant reservoirs of bat populations. The island boasts a high diversity of bat species, with over 200 species (constituting 20% of the World’s bat species) found across Indonesia. Of these, 75 species inhabit Sulawesi. This distinctiveness has established Sulawesi as the island with the world’s highest diversity of fruit bats and a remarkable diversity of endemic species [66, 67]. The presence of Sulawesi-endemic species such as *Dobsonia viridis*, *Rousettus celebensis*, and *Styloctenium wallacei*, which have been confirmed to be positive for the JE virus, is a critical factor that warrants attention. Bats can be found in all provinces in Sulawesi, but there are still cities/regencies that are not yet known because research is usually carried out in certain places that are close to nature reserves, tourist attractions, or that have their own issues, such as bat markets. The Sulawesi bats that have been reported consist of seven families, i.e., *Pteropodidae*, *Megadermatidae*, *Vespertilionidae*, *Rhinolophidae*, *Hipposideridae*, *Emballonuridae*, and *Molossidae*, with 32 genera (some species in Figure 3) [68]. Bats in Sulawesi have already been reported as reservoirs for JE, namely *D. viridis*, *R. celebensis*, *Rousettus amplexicaudatus*, *S. wallacei*, *Toopterus nigrescens*, *Kerivoula hardwickii*, and *Myotis ater* [69, 70]. The reported Ardeid birds and bat species in Sulawesi are shown in Table 1 [71–114].

**Table 1:** Ardeid bird and bat species reported in Sulawesi.

Regency/City	Ardeid bird	References	Bat	References
Gorontalo				
Boalemo			*Acerodon* spp. *Pteropus alecto*	[71]
Bone Bolango	*Ixobrychus sinensis, I. cinnamomeus, I. flavicollis, Nycticorax caledonicus, Butorides striata, Ardeola speciosa, Bubulcus ibis, Ardea sumatrana, A. purpurea, A. alba, A. intermedia, Egretta garzetta, E. sacra*	[72]	*Acerodon celebensis, Pteropus alecto, and Pteropus* spp.	[73]
West Sulawesi				
Mamuju	*Ardeola speciosa, Ardea purpurea, Egretta garzetta, Ixobrychus cinnamomeus*	[74]	-	
Polewali Mandar	*Egretta garzetta, Butorides striata, Gorsachius melanolophus, Ardea sumatrana, A. purpurea, Ardeola speciosa, Bubulcus ibis, Nycticorax nycticorax, Egretta alba, Nycticorax caledonicus*	[75,76]	-	
South Sulawesi				
Barru	*Ardea sumatrana, Ardeola speciosa, Butorides striata, and Egretta garzetta nigripes*	[77]	*Eonycteris spelaea, Rousettus amplexicaudatus, R. celebensis, Hipposideros* spp.	[78–80]
Bone	*Ardea purpurea, Egretta eulophotes, E. garzetta, and Nycticorax caledonicus*	[76,81]		
Bulukumba			*Acerodon celebensis, Harpyionycteris celebensis, Macroglossus minimus, Nyctimene cephalotes, Styloctenium wallacei., Thoopterus nigrescens., Cynopterus minutus, Cynopterus* cf. *sphinx, Dobsonia exoleta, Dobsonia viridis, Dobsonia* cf. *viridis, Eonycteris spelaea, Pteropus Alecto, Rousettus amplexicaudatus, Rousettus celebensis, Rhinolophus philippinensis, Scotophilus kuhlii*	[82]
Enrekang	*Egretta alba, Ardeola speciosa, and Ixobrychus sinensis*	[76,83]		
Gowa			*Thoopterus nigrescens, Thoopterus suhaniahae, Boneia bidens, Dobsonia* spp.*, Rousettus* spp.	[84, 85]
Jeneponto	*Bubulcus ibis*	[76]	-	
Kepulauan Selayar	*Ardea purpurea, Butorides striata, and Egretta sacra*	[86]	-	
East Luwu	*Ixobrychus flavicollis*	[76]	*Macroglossus minimus., Nyctimene cephalotes, Thoopterus nigrescens, Cynopterus brachyotis, Dobsonia exoleta, Dobsonia viridis, Eonycteris spelaea, Pteropus Alecto, Rousettus amplexicaudatus, Rousettus celebensis, Megaderma spasma, Phoniscus* cf. *jagori Rhinolophus philippinensis*	[82]
Makassar	*Egretta eulophotes*	[76]	*Acerodon* spp. *and Rousettus* spp.	(Unpublish)
Maros	*Ixobrychus sinensis and Ixobrychus flavicollis*	[76]	*Hipposideros* spp.*, Rhinolophus* spp.*, Acerodon* spp.*, Myotis* spp.*, Acerodon* spp.	[87–89]
Palopo	*Ardea sumatrana, Nycticorax caledonicus, N. nycticorax, and*	[76]	-	
Pangkep	*Ixobrychus flavicollis, Ixobrychus flavicollis australis (gouldi)*	[76]	*Hipposideros* spp.*, Rhinolophus* spp.*, Myotis* spp.*, Acerodon celebensis., Macroglossus minimus., Styloctenium wallacei., Cynopterus brachyotis, Cynopterus* cf. *sphinx., Dobsonia viridis, Dobsonia* cf. *minor Eonycteris major, Eonycteris spelaea., Pteropus* spp.*, Pteropus* cf. *hypomelanus, Rousettus amplexicaudatus, Rousettus celebensis, Rousettus* cf. *bidens, Hipposideros* spp.*, Myotis adversus, Pipistrellus* spp.	[82, 87, 89]
Pare Pare	*Bubulcus ibis*	[76]	-	
Pinrang	*Ixobrychus flavicollis*	[76]	-	
Sidrap	*Egretta eulophotes, Ardea purpurea, and Bubulcus ibis*	[81]	-	
Soppeng			*Pteropus vampirus, Rousettus* spp.*, Acerodon* spp.	[90]
Wajo	*Egretta eulophotes, Ardea purpurea Nycticorax caledonicus, and Ixobrychus flavicollis*	[76,81]	-	
Central Sulawesi				
Donggala	*Bubulcus ibis*	[76]	-	
Palu	*Scolopax celebensis, Ardea sumatrana*	[91,92]	-	
Morowali	*Ardea sumatrana*	[76]	*Rousettus celebensis, Acerodon celebensis, Pteropus alecto*	[73]
North Morowali	*Nycticorax nycticorax*	[76,93]	-	
Poso	*Ardea purpurea, Ardeola speciosa, Bubulcus indicus, Egretta intermedia, E. garzetta, E. alba, Ixobrychus flavicollis*		*Pteropus* spp.	[73]
Sigi			*Rousettus celebensis, Rousettus linduensis Macroglossus minimus.*	[94]
Tojo Una-Una	*Butorides striata, Egretta garzetta, A. sumatrana, A. purpurea, Ixobrychus cinnamomeus*	[95]	-	
Tolitoli	*Ardea sumatrana and Ixobrychus flavicollis*	[76]	-	
Southeast Sulawesi				
Bombana	*Ixobrychus cinnamomeus, I. flavius, Bubulcus ibis, E. Garzetta, E. Alba, N. caledonicus, N. Nycticorax, Ardea purpurea, Ardeola speciosa, Ardea sumatrana, Casmerodius albus, Egretta intermedia, Nycticorax caledonicus, Ixobrychus sinensis, Ixobrychus cinnmomeus, Ixobrychus flavicollis*	[96,97]	*Cynopterus brachyotis, Cynopterus* cf. *brachyotis, Cynopterus* cf. *minutus, Cynopterus* spp.*, Cynoptherus* cf. *brachiotis, Dobsonia* cf. *exoleta, Dobsonia exoleta, Dobsonia* spp.*, Dobsonia viridis, Eonycteris* cf. *spelaea, Eonycteris spelaea, Hipposideros* cf. *Cineraceus, Hipposideros* spp. *Kerivoula* spp. *Macroglossus minimus, Macroglossus sobrinus, Mops* spp.*, Myotis* spp.*, Nyctimene cephalotes, Nyctimene* cf. *cephalotes, Pipistrellus* spp.*, Pteropus Alecto, Rousettus amplexicaudatus, Rousettus celebensis, Rousettus* cf. *amplexicaudatus, Styloctenium wallacei, Thoopterus nigrescens*	[70]
Buton			*Chironax* spp.*, Cynopterus* spp. *macroglosus, Nyctimene, Rousettus, Thoopterus, Emballonura, Mosia, Hipposideros, Rhinolophus, Megaderma, Kerivoula, Miniopterus, Myotis, and Philetor*	[98, 99]
South Buton	*Ardea purpurea,* *A. novaehollandiae, Egretta garzetta, Butorides striatus, E. sacra, and ixobrychus cinnamomeus*	[100]	-	
Kolaka			*Chironax, Cynopterus, macroglosus, Nyctimene, Rousettus, Thoopterus, Emballonura, Mosia, Hipposideros, Rhinolophus, Megaderma, Kerivoula, Miniopterus, Myotis, Philetor*	[98]
East Kolaka	*Ixobrychus cinnamomeus, I. flavius, Bubulcus ibis, E. Garzetta, E. Alba, N. caledonicus, N. Nycticorax, Ardea purpurea, Ardeola speciosa, Ardea sumatrana, Casmerodius albus, Egretta intermedia, Nycticorax caledonicus, Ixobrychus sinensis, Ixobrychus cinnmomeus, Ixobrychus flavicollis*	[96, 97]	-	
Konawe	*Ardea intermedia, A. alba,* *A. sumatrana, A. purpurea, Ardeola speciosa, Bubulcus ibis, Egretta garzetta, Egretta alba, Egretta intermedia, Nycticorax caledonicus, N. nycticorax, Casmerodius albus, Nycticorax caledonicus, Egretta garzetta, Bubulcus ibis, Ixobrychus cinnamomeus, Ixobrychus flavicollis, Ixobrichus sinensis*	[96,97,101]	*Chaerephon plicatus, Cynopterus brachyotis, Cynopterus* cf. *brachyotis, Cynopterus minutus, Dobsonia* spp.*, Dobsonia viridis, Eonycteris spelaea, Hipposideros* spp.*, Macroglossus minimus, Megaderma spasma, Myotis* spp.*, Nyctimene cephalotes, Pteropus alecto, Pteropus* cf. *Alecto, Rhinolophus* spp.*, Rousettus amplexicaudatus, Rousettus celebensis, Styloctenium wallacei*	[70]
Konawe Kepulauan	*Egretta garzetta, A. intermedia,* *A. alba, A. purpurea, and Butorides striata*	[101,102]	*Thoopterus shanahan*	[99]
South Konawe	*The same applies to Bombana.*	[96,97]	-	
Kendari			*Chironax melanocephalus, Cynopterus luzoniensis, Macroglossus minimus, Nyctimene cephalotes, Rousettus celebensis, Thoopterus nigrescens, Emballonura alecto, Mosia nigrescens, Hipposideros cervinus, Hipposideros pelingensis, Hipposideros boeadii, Rhinolophus arcuatus, Rhinolophus philippinensis, Rhinolophus celebensis, Rhinolophus euryotis, Megaderma spasma, Kerivoula hardwickii, Miniopterus fuliginosus, Myotis* cf. *ridleyi, Philetor brachypterus*	[98]
Muna	*Ardea sumatrana, Ardea purpurea, and Butorides striata*	[101]	*Cynopterus brachyotis, Cynopterus minutus, Dobsonia* cf. *crenulate, Dobsonia viridis, Eonycteris spelaea, Kerivoula hardwickii, Macroglossus minimus, Nyctimene cephalotes, Pipistrellus* spp.*, Pteropus alecto, Rousettus amplexicaudatus, Rousettus celebensis, Styloctenium wallacei*	[70]
North Sulawesi				
Bitung	*Ardea sumatrana, Egretta sacra, Nycticorax nycticorax, and Gorsachius goisagi*	[76]	*Acerodon celebensis, Cynopterus brachyotis, Dobsonia exoleta, Eonycteris spelaea, Kerivoula hardwickii, Macroglossus minimus, Nyctimene cephalotes, Rousettus amplexicaudatus, Rousettus celebensis, Thoopterus*	[69, 103, 104, 105]
			*nigrescens, Boneia bidens, Megaderma spasma, Rhinolophus celebensis*	
Bolaang Mongondow	*Ixobrychus sinensis,* *I. cinnamomeus, I. flavicollis, Nycticorax caledonicus, Butorides striata, Ardeola speciosa, Bubulcus ibis, Ardea sumatrana, A. purpurea, A. alba, A. intermedia, E. garzetta, Ardea sumatrana, Egretta sacra*	[72,76]	*Acerodon celebensis, Dobsonia exoleta, Neopteryx frosti, Styloctenium wallacei, Rousettus amplexicaudatus, Rousettus* spp.*, Thoopterus nigrescens, Nyctimene cephalotes, Cynopterus minutus*	[106, 107]
South Bolaang Mongondow	*Ixobrychus sinensis,* *I. cinnamomeus, I. flavicollis, Nycticorax caledonicus, Butorides striata, Ardeola speciosa, Bubulcus ibis, Ardea sumatrana, A. purpurea, A. alba, A. intermedia, E. garzetta, Ardea sumatrana Egretta sacra*	[72,76]	-	
North Bolaang Mongondow	*Ixobrychus sinensis,* *I. cinnamomeus, I. flavicollis, Nycticorax caledonicus, Butorides striata, Ardeola speciosa, Bubulcus ibis, Ardea sumatrana, A. purpurea, A. alba, A. intermedia, E. garzetta, Ardea sumatrana Egretta sacra*	[72,76]	-	
Kepulauan Sangihe	*Egretta alba, E. intermedia,* *E. Gazetta, E. Sacra, Bubulcus ibis, Butorides striatus*	[108]	-	
Kepulauan Talaud	*Egretta alba, E. intermedia,* *E. Gazetta, E. Sacra, Bubulcus ibis, Butorides striatus, Nycticorax caledonicus, Gorsachius melanolophus*	[108]	-	
Manado	*Ardea purpurea* *Gorsachius goisagi*	[76,109]	*Acerodon celebensis, Cynopterus brachyotis, Thoopterus nigrescens, Macroglossus minimus, Nyctimene cephalotes, Rousettus amplexicaudatus, Pteropus alecto,*	[69, 73]
Minahasa	*Bubulcus ibis, Ardeola speciosa* *Egretta intermedia* *Egretta garzetta, E. alba. Bubulcus ibis, Nycticorax nycticorax, Ixobrichus sinensis, Ardea purpurea, Egretta alba, Egretta intermedia, Egretta garzetta, Bubulcus ibis, Ardeola speciosa, Butorides striatus, Nycticorax caledonicus, Ixobrychus sinensis, I. cinnamomeus,* *I. flavicolis,*	[76,110,111]	*Cynopterus luzonensis, Cynopterus sphinx, Cinopterus brachyotis, Macroglossus minimus, Rhinolophus, Rousettus celebensis, Rousettus amplexicaudatus Myotis ater, Acerodon celebensis, Dobsonia exoleta, Neopteryx frosti, Styloctenium wallacei, Thoopterus nigrescens, Nyctimene cephalotes*	[69, 107]
South Minahasa			*Dobsonia exoleta, Nyctimene cephalotes, Rousettus amplexicaudatus, Rousettus celebensis, Thoopterus nigrescens, Cynopterus minutus*	[73, 112]
North Minahasa			*Rousettus amplexicaudatus, Rousettus celebensis, Nytimene cephalotes, Cynopterus minutus, Thoopterus nigrescens*	[113]
Tomohon	*Bubulcus ibis, Ardea purpurea*	[114]		

*E. garzetta*, *A. alba*, and *B. ibis* are species that are found in almost all swamp or coastal ecosystems in Sulawesi. This represents great potential as well as a challenge to determine their role and patterns in JE transmission. Birds are usually found in large numbers and inhabit areas for a long time. In addition, bats known as JE reservoirs, such as *D. viridis*, *R. celebensis*, and *R. amplexicaudatus*, are also often found in Sulawesi. These two factors are the driving force and the possibility of maintaining the JE virus cycle in nature.

Birds are highly susceptible to seasonal and environmental factors. Certain species dominate specific areas under favorable environmental conditions, whereas they decline in highly polluted areas. Furthermore, bird activity rises during the rainy season because of the need for feeding, perching, and breeding, and it generally declines during the dry season [115, 116]. Species of the Ardeidae are also dependent on aquatic macrophytes, so their presence is always associated with the ecological conditions of water bodies [117].

Similarly, the presence of bats around human settlements depends on their food sources. Bats usually remain in one area where they rest, which differs from where they forage. In general, bats are not disturbed by the presence of humans, although some species may be affected by changes in human activities, such as differences in weekdays and weekends [118].

The JE virus requires the mosquito vector to be transmitted to other hosts. There are at least 10 species of mosquitoes that are potential vectors in Indonesia and can be found spread across Sulawesi, as presented in Table 2 [14, 15, 69, 70, 82, 119–137].

**Table 2:** Potential JE mosquito vectors in Sulawesi.

Regency/City	Mosquito species code								

	A	B	C	D	E	F	G	H	I
Gorontalo									
Gorontalo		Hamzah [119]							
Mamuju		Nurdin *et al*. [120]							
Pasangkayu	Rahma *et al*. [121]	Rahma *et al*. [121]	Rahma *et al*. [121]	Rahma *et al*. [121]		Rahma *et al*. [121]	Rahma *et al*. [121]	Rahma *et al*. [121]	Rahma *et al*. [121]
Polewali Mandar		Davidson *et al*. [122]							
South Sulawesi									
Bulukumba	Ministry of Health [82]	Ministry of Health [82], Tilka and Ridjal [123]	Ministry of Health [82]	Ministry of Health [82]	Ministry of Health [82]	Ministry of Health [82]	Ministry of Health [82]	Ministry of Health [82]	Ministry of Health [82]
Enrekang	Maksud *et al*. [124]	Maksud *et al*. [124], Lien *et al*. [125]	Maksud *et al*. [124]	Maksud *et al*. [124]	Maksud *et al*. [124]	Maksud *et al*. [124]	Maksud *et al*. [124]	Maksud *et al*. [124]	Maksud *et al*. [124]
East Luwu	Ministry of Health [82]	Ministry of Health [82], Lien *et al*. [125]		Lien *et al*. [125]	Ministry of Health [82], Lien *et al*. [125]	Ministry of Health [82], Lien *et al*. [125]	Ministry of Health [82], Lien *et al*. [125]	Ministry of Health [82]	Ministry of Health [82]
Makassar		Lien *et al*. [125], Karmila *et al*. [126], Rahma *et al*. [127], Davidson *et al*. [128]		Karmila *et al*. [126]	Lien *et al*. [125], Karmila *et al*. [126], Davidson *et al*. [128]	Lien *et al*. [125], Karmila *et al*. [126]	Lien *et al*. [125], Karmila *et al*. [126]	Karmila *et al*. [126]	Karmila *et al*. [126]
Maros	Rahma *et al*. [121]	Rahma *et al*. [121], Davidson *et al*. [128]	Rahma *et al*. [121]	Rahma *et al*. [121]	Davidson *et al*. [128]	Rahma *et al*. [121]	Rahma *et al*. [121]	Rahma *et al*. [121]	Rahma *et al*. [121]
Palopo							Lien *et al*. [125]		
Pangkep	Ministry of Health [82], Setiyaningsih *et al*. [129]	Ministry of Health [82], Setiyaningsih *et al*. [129]		Ministry of Health [82], Setiyaningsih *et al*. [129]	Ministry of Health [82], Setiyaningsih *et al*. [129]	Ministry of Health [82], Setiyaningsih *et al*. [129]	Ministry of Health [82], Setiyaningsih *et al*. [129]	Ministry of Health [82], Setiyaningsih *et al*. [129]	Ministry of Health [82], Setiyaningsih *et al*. [129]
North Toraja	Rahma *et al*. [121]	Lien *et al*. [125], Rahma *et al*. [121]	Rahma *et al*. [121]	Rahma *et al*. [121]	Lien *et al*. [125], Rahma *et al*. [121]	Rahma *et al*. [121]	Rahma *et al*. [121]	Rahma *et al*. [121]	Rahma *et al*. [121]
Central Sulawesi									
Donggala		Wigati and Maksud [15], Fahmi *et al*. [130], Sabir *et al*. [131]	Sabir *et al*. [131], Mogi *et al*. [132]	Mogi *et al*. [132]		Sabir *et al*. [131]		Sabir *et al*. [131]	Sabir *et al*. [131]
Palu									Mogi *et al*. [132]
Parigi Moutong	Garjito *et al*. [133]	Garjito *et al*. [133]							
Sigi		Udin *et al*. [134]							
Southeast Sulawesi									
Bombana		B2P2VRP [70]	B2P2VRP [70]	B2P2VRP [70]	B2P2VRP [70]	B2P2VRP [70]	B2P2VRP [70]	B2P2VRP [70]	B2P2VRP [70]
North Buton		Lempang *et al*. [14]		Podung *et al*. [135]	Podung *et al*. [135]		Podung *et al*. [135]	Podung *et al*. [135]	
Konawe	B2P2VRP [70]	B2P2VRP [70]	B2P2VRP [70]	B2P2VRP [70]	B2P2VRP [70]	B2P2VRP [70]	B2P2VRP [70]	B2P2VRP [70]	
Muna		B2P2VRP [70]	B2P2VRP [70]	B2P2VRP [70]	B2P2VRP [70]	B2P2VRP [70]	B2P2VRP [70]	B2P2VRP [70]	B2P2VRP [70]
West Muna								Linarsih *et al*. [136]	
North Sulawesi									
Bitung		B2P2VRP [69]	B2P2VRP [69]	B2P2VRP [69]	B2P2VRP [69]	B2P2VRP [69]	B2P2VRP [69]	B2P2VRP [69], Eman *et al*. 137]	B2P2VRP [69]
Manado	B2P2VRP [69]	B2P2VRP [69]	B2P2VRP [69]	B2P2VRP [69]	B2P2VRP [69]	B2P2VRP [69]	B2P2VRP [69]	B2P2VRP [69]	B2P2VRP [69]
Minahasa	B2P2VRP [69]	B2P2VRP [69], Podung *et al*. [135]	B2P2VRP [69], Mogi *et al*. [132]	B2P2VRP [69]	B2P2VRP [69], Podung *et al*. [135]	B2P2VRP [69], Mogi *et al*. [132], Podung *et al*. [135]	B2P2VRP [69], Mogi *et al*. [132], Podung *et al*. [135]	B2P2VRP [69], Podung *et al*. [135]	B2P2VRP [69], Mogi *et al*.[132] Podung *et al*. [135]
Tomohon					Podung *et al*. [135]	Podung *et al*. [135]	Podung *et al*. [135]	Podung *et al*. [135]	Podung *et al*. [135]

A=*Anopheles kochi*, B=*Anopheles vagus*, C=*Armigeres subalbatus*, D=*Culex bitaenhyorinchus*, E=*Culex fuscocephala*, F=Culex gelidus, G=*Culex tritaenhyorinchus*, H=*Culex quinquefasciatus*, I=*Culex vishnui*, JE=Japanese enchepalitis

Vector species seem to tend to reproduce in the same ecosystem, except for *A. kochi*, *A. subalbatus*, and *C. fuscocephala*. This suggests that several species can be found together, such as *C. tritaeniorhynchus* and *C. vishnui*. In addition, *A. annularis* appears to have never been reported in Sulawesi.

## JE REPORTS IN SULAWESI

Sulawesi has many cases of fever known as “step,” which is referred to as “febrile convulsions.” The cause may be due to various reasons, but it indicates an undiagnosed case of JE diseases. Unfortunately, JE screening is not a routine government program, such as tuberculosis, AIDS, leprosy, malaria, and dengue fever screening, and the effect is that there are no annual data [59–64]. The reports have only been published by research groups and institutions since 1972. The Ministry of Health’s community-based surveillance program was conducted in Bali in 2001–2003 and then developed into the Sentinel JE Surveillance System (S3JE) program in 2014 in five provinces, and in 2016, it was developed into eleven provinces, including North Sulawesi [138]. The reservoir study was conducted through national-scale research using the term “Rikhus Vektora” (Riset Khusus Vektor dan Reservoir Penyakit or Special Research on Disease Vectors and Reservoirs). The JE detection report in Sulawesi is presented in Table 3 [36, 69, 70, 129, 139–146].

**Table 3:** JE reported in Sulawesi.

Province	Regency/City	Types of sample	Year		Detection	References

			Sampling	Report		
South Sulawesi	East Luwu	Human	1973	2016	HI	[139]
	Makassar	Human	1972	1979	NA	[140]
	Maros	Cattle, pig, chicken, and goat	1996 –1997	2000	Competitive ELISA	[36]
	Pangkep	Mosquito	2017	2017	PCR	[129, 141]
	Tana Toraja	Pig	2012	2015	ELISA	[142]
Central Sulawesi	Sigi	Pig	2012	2015	ELISA	[142]
Southeast Sulawesi	Kolaka	Human	1972	1979	NA	[140]
	Muna	Ba	2016	2016	PCR	[70, 143]
North Sulawesi	Bitung	Bat	2016	2016	PCR	[69]
	Manado	Human	2014	2016	IgM Capture ELISA	[144]
			2015 –2017	2018	Anti-JEV IgMELISA	[145]
		Bat	2016	2016	PCR	[69]
	Minahasa	Pig	-	2021	Competitive ELISA	[146]
		Bat	2016	2016	PCR	[69]
	South Minahasa	Pig		2021	Competitive ELISA	[146]
	Tomohon	Pig	2012	2015	ELISA	[142]
				2021	Competitive ELISA	[146]

HI=Hemagglutination Inhibition, NA=Neutralizing Antibody, PCR=Polymerase chain reaction, ELISA=Enzyme-linked immunosorbent assay, IgM=Immunoglobulin M, JE=Japanese encephalitis

**Figure 4:**
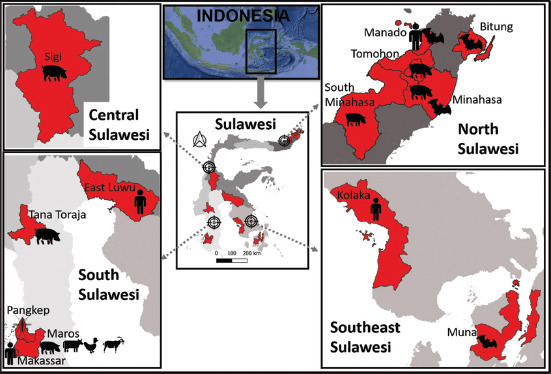
Map of Sulawesi showing locations with reported JE virus presence (red). Icons indicate sample types: human, pig, bat, cattle, chicken, goat, and mosquito [Source: https://tanahair.indonesia.go.id; Map processed using QGIS version 3.40.3-Bratislava, https://qgis.org].

Cases of JE in humans in Sulawesi have been reported in four cities/districts in Sulawesi (Figure 4). Some of these reports are from the past (since 1972), and recent data are lacking. The lack of data may be due to the lack of tools to detect JE, although in Sulawesi, there are many cases with symptoms of encephalitis, which is one of the JE syndromes. Positive samples for JE in humans were first detected in serum samples in 1972 from Ujung Pandang (Ex-Makassar) and Pomalaa (Kolaka) using a neutralizing antibody test and in 1973 from Malili (East Luwu) using hemagglutination inhibition (HI) [139, 140]. In subsequent research, human cases were found in Manado in 2014, and in 2015–2017, a case study was carried out on 74 patients with clinical symptoms of the CNS in the same city using the ELISA test. The results showed that JE examination was low (1.6%) [144, 145]. In this study, the samples were from adult patients, which may be the reason why the seropositivity rate was low, considering that JE cases are more common in children.

JE virus antibodies were detected in pigs in Maros (samples collected in 1996–1997) and in 2012 samples from Tana Toraja, Sigi, and Tomohon [36, 142]. The last report in 2021 (sampling year not stated) JE in pigs was in Minahasa, South Minahasa, and Tomohon [146]. Other reservoirs, including goats, cattle, and chickens, were detected from Maros using the ELISA test [36] (Figure 4). Positive samples in bats have been reported from national scale studies (Rikhus Vectora) by polymerase chain reaction (PCR) tests in 2016 and 2017, such as in *D. viridis*, *R. celebensis*, and *S. wallacei* from Muna [70, 143]; *T. nigrescens* and *R. amplexicaudatus* in Manado; *K. hardwickii* and *R. celebensis* in Bitung; and in Minahasa from *R. celebensis* and *M. ater* [69]. The JE virus-positive mosquito vector was detected only in Pangkep, specifically in *Cx. tritaeniorhynchus*, through PCR testing [129, 147].

## THE CURRENT PERSPECTIVE ON JE IN SULAWESI

Based on historical reviews, risk factors are present in almost all areas of Sulawesi. This finding is in contrast with the JE disease data. The discrepancy is influenced by various factors, including the host, vector, detection tools, environment, and socioeconomic conditions. The pig population in Sulawesi is high, especially in South Sulawesi, which has the second largest pig population in Indonesia after East Nusa Tenggara in the past 3 years [148]. Almost all areas in Sulawesi have pig farms. Although there are no data from statistical agencies, pigs are usually kept “hidden” around residential areas, even in urban centers. However, pigs tend to be concentrated in non-Muslim areas because they are considered forbidden by Muslims. In Sulawesi, the largest pig populations are found in North Toraja, Tana Toraja, and Mamasa (Table 1) [71–114].

JE research on the Ardeid family in Sulawesi has not been conducted. However, data on Ardeid birds throughout Sulawesi province indicate a high level of alertness. This species can be found in Sulawesi, especially in coastal rivers, ponds, and swamps. Birds are also a consumption choice for the community, although they are not as prevalent as other poultry, such as chickens and ducks.

The presence of reservoirs such as bats does not seem to have a significant impact, as although bats can be found, the chances of transmission are low. This is influenced, for example, by bats, which are considered sacred and thus are not disturbed by the community. Hunting of bats for meat consumption does occur but is limited to certain areas, similar to pork consumption. On the other hand, not all bat species are dangerous and have many functions in the ecosystem, but they still need to be monitored. Especially bats that have previously been confirmed as reservoirs for certain viruses. Considering previous cases of the COVID-19 pandemic, numerous factors that may be the primary cause of the virus outbreak warrant consideration.

In addition to bats, positive samples of the JE virus were also found in livestock, especially in cows, goats, and chickens. After that, there was no further research, and this is an important concern, especially because livestock are around human settlements. Knowledge of the multianimal host JE reservoir is necessary. Birds, poultry, and other livestock are abundant in Sulawesi and have a role in many benefits, such as food sources, transportation, and pets; therefore, they need to be studied further. The role of species must be considered in terms of potential risks, even though they are not directly related to the transmission of JE disease [149]. Understanding the host is related to its transmission ability and the genotype of the virus being transmitted. Different hosts may transmit different genotypes; for example, the JE GI virus replicates more efficiently in duck and chicken cells than the JE GIII virus, thus affecting the distribution of genotypes distributed [10].

Knowledge of JE in mosquito vectors in Sulawesi is still limited, with only one study [129] to date, namely *Cx. tritaeniorhynchus* from Pangkep (Pangkajene and the Islands) in 2017. This species is the main vector for transmitting JE, and it has extensive breeding areas, especially rice fields. Other species cannot be ignored, including *Cx. gelidus, Cx. vishnui, Cx. fuscocephala, Cx. bitaeniorhynchus, An. vagus, An. kochi, and* *An. annularis*, which can be found together with *Cx. tritaeniorhynchus*.

The tropical climate makes agriculture the main livelihood in rural areas and even in city centers; therefore, rice fields can be found in all provinces [150]. However, the activities of farmers who cultivate seasonal rice fields each year appear to provide other benefits. Mosquitoes breed during the rainy season, but their survival rate decreases as the harvest season approaches, leading to a drastic reduction in their production. This cycle occurs simultaneously in almost all productive rice fields, collectively affecting the life of vectors. In addition, rivers and swamps are also potential breeding sites for *An. vagus*. Other water sources include fragmented grasslands, waterways, and accumulated hydrological runoff [35, 151]. It seems that this balance will remain in nature.

The presence of mosquitoes around humans depends on their blood-sucking sources. *Cx. tritaeniorhynchus* is zooanthropophilic (bloodmeal from human and animal) so its existence is greatly influenced by the presence of livestock, including pigs. In this condition, the presence of livestock increases the number of mosquitoes, especially zoophilic and zooanthropophilic mosquitoes. Unlike *Cx. quinquefasciatus*, which is anthropophilic, its existence is strongly influenced by the number of people in the house [152]. Recent research shows that *Cx. vishnui, Cx. sitiens, and Cx. bitaeniorhynchus* are ornithophilia (although it also bites humans), in contrast to *Cx. gelidus* and *Cx. quinquefasciatus*, which prefer mammals [153]. Other studies have shown that *Cx. vishnui, Cx. gelidus, and* *Cx. tritaeniorhynchus* are more interested in pigs than cows, whereas cows are also a favorite even though they are lower [154].

Research on mosquitoes as JE vectors is still lacking, compared with the vectors of other diseases, such as dengue fever, filariasis, and malaria. However, mosquitoes can also play a dual role in transmitting disease. For example, the malaria vector *An. vagus* can also transmit the JE virus, and *Cx. quinquefasciatus* serves as a vector for JE and filarial diseases. The ability of one mosquito species to carry multiple viruses poses a challenge to human and animal health. Therefore, other mosquito species need to be considered as well, especially if they also carry other viruses, such as *Aedes albopictus*, which is known as a vector of chikungunya and dengue fever but also plays a role in the transmission of JE [155].

Despite these factors, the most influential aspect of the data reports is the lack or insufficiency of diagnostic kits for JE detection. As a result, most reported fever cases are only classified as common neurological cases and are not associated with the JE virus. In fact, JE disease in Sulawesi might be considered a neglected disease, but if new cases are found, they are considered emerging cases.

It cannot be denied that the development of globalization can increase the risk factors for JE transmission, including economic, cultural, and environmental factors. For example, an economic issue is a large number of wild animal meat trade activities in North Sulawesi (31 markets regularly sell wild animal meat such as pythons, rats, wild boars, dogs, and bats) [156–159]. In cultural settings, many traditional ceremonies or celebrations use pork as an obligatory food. For example, in Tana Toraja and North Toraja, it is customary to consume pork at every traditional event [160, 161]. Learning from Bali shows the potential for transmission in several areas where there are many pigs, such as Manado, North Toraja, and Tana Toraja [162].

Environmental factors are related to demographics and climate change. This is a major challenge in the 21^st^ century, even though participation and digitalization are necessary. The impact of environmental change can be seen in outbreaks of zoonotic and vector-borne diseases, which have increased on average from 1990 to 2016 and are linked to deforestation, especially in tropical countries such as Indonesia [163, 164]. Climate change is another factor that supports the transmission cycle. Global warming will increase the bionomic competence of vectors, which has a direct impact on transmission. The pattern of JE transmission is strongly influenced by the season, which increases in the rainy season when the transmission vector increases. Climate change can influence transmission trends because the cycle is naturally influenced by the annual mean temperature (AMT). The optimal temperatures for the JE mosquito vector are 22.8°C–34.5°C. High temperatures increase the reproduction of mosquito larvae, thereby shortening the incubation period for virus replication in the mosquito body and accelerating the transmission of the virus from mosquitoes to humans or animals [151, 165].

JE data positive in Sulawesi shows that several areas have become endemic, including North and South Sulawesi, although only a few human cases have been detected. To date, no antiviral drugs have been approved by the Food and Drug Administration for treating JE [166]. Efforts made to find drugs are, for example, related to the search for JE virus-specific monoclonal antibodies; the identification of natural medicinal plant products by investigating absorption, distribution, metabolic processes, excretion, and toxicity; and the search for viral protease inhibitors and host-directed antiviral agents. This is particularly challenging because of possible changes in vector distribution and pathogen epidemiology [167, 168]. Until now, no vaccine has been administered in Sulawesi. Vaccines are being promoted in Indonesia in Bali [169] and West Kalimantan [170].

The development of virus detection methods using neutralizing antibodies and HI tests for ELISA and PCR methods is growing rapidly. This is beneficial for researchers seeking accurate results using a relatively fast method. The proposed method also needs to be considered between costs and expected results. In the future, initial surveillance can be carried out using ELISA and PCR methods, followed by culturing to determine the genomics of the virus [171].

## CONCLUSION

This study provides an in-depth historical review and contemporary analysis of JE in Sulawesi, Indonesia, highlighting its ecological, sociocultural, and epidemiological dynamics. The findings underscore the significant risk posed by the presence of amplifying hosts, such as pigs and waterfowl, the abundance of mosquito vectors, particularly *Cx. tritaeniorhynchus*, and the widespread reservoirs, including endemic bats and birds. Sulawesi’s geographic and environmental characteristics, combined with its cultural practices, create an ideal setting for JE transmission, yet the lack of consistent government-led JE surveillance programs has left the true prevalence of the disease largely underreported.

The study’s strength lies in its comprehensive approach, integrating historical data with contemporary ecological and epidemiological insights. It identifies risk factors across hosts, vectors, and environmental contributors while emphasizing the cultural and economic dimensions unique to Sulawesi. In addition, the study compiles multihost species data, offering a holistic view of JE’s complex transmission dynamics. This evidence-based synthesis serves as a valuable reference for policymakers, researchers, and public health practitioners.

However, the review is constrained by the sporadic and fragmented availability of JE-related data in Sulawesi. A lack of routine diagnostic surveillance and underreporting by health authorities limits the ability to estimate the disease burden accurately. Furthermore, data from independent research efforts may not be representative of all Sulawesi regions, creating potential gaps in the understanding of JE’s true geographic spread and seasonal patterns.

To address these limitations, future studies should focus on systematic surveillance programs incorporating molecular and serological tools, alongside GIS-based mapping of high-risk areas. Research on vector competence, host-specific viral dynamics, and genotype distributions across various reservoirs is critical to understanding JE’s ecological nuances. In addition, assessing the feasibility and impact of community-based vaccination programs and enhancing public awareness campaigns in high-risk regions could significantly reduce disease incidence. As climate change alters vector populations and disease cycles, integrating climate modeling with JE studies will also be imperative.

In conclusion, this study highlights the urgent need for targeted interventions and collaborative research to combat JE in Sulawesi, ensuring sustainable public health outcomes in the region.

## AUTHOR’S CONTRIBUTIONS

All authors contributed to the conception and design of this review. NR: Drafted the manuscript; analyzed data, figures, tables; and edited the manuscript. HN, NID, IS, RSA, MS, and RR: Guided the writing flow and reviewed the manuscript. IW: Supervised the review, guided the writing flow, reviewed the draft, and updated figures and tables. All authors have read and approved the final manuscript.
